# Expert committee declares WHO Region of the Americas measles-free

**DOI:** 10.2807/1560-7917.ES.2016.21.39.30360

**Published:** 2016-09-29

**Authors:** Eurosurveillance editorial team

**Affiliations:** 1 European Centre for Disease Prevention and Control (ECDC), Stockholm, Sweden

The World Health Organization (WHO) Region of the Americas has been declared measles-free.

During a Pan American Health Organisation (PAHO)/WHO meeting on 27 September 2016 the International Expert Committee (IEC) for Documenting and Verifying Measles, Rubella, and Congenital Rubella Syndrome Elimination in the Americas announced that measles has been eliminated from the region. This follows a campaign which has lasted 22 years since efforts started in 1994, when the 35 countries of the Region launched an initiative to eliminate measles, rubella and congenital rubella syndrome.

The road to measles elimination has not been smooth. Measles transmission in the Region had been considered interrupted since 2002, when the last endemic case was reported in the Americas. Between 2003 and 2010, there was an average of 153 cases annually, either imported or linked to imported cases. However, large outbreaks between 2011 and 2015 in Brazil, Canada, Ecuador, and the United States resulted in 8–12 times more reported cases than in the preceding period. Brazil was the last country of the Region to experience a prolonged measles outbreak, from 2013 to 2015 [1]. In April 2015, the IEC declared that endemic measles transmission had emerged in Brazil. Brazil considered that measles transmission ended in July 2015, after the last confirmed case had been reported on 13 June 2013 [2]. The IEC reviewed evidence on measles elimination presented by all the countries of the Region between 2015 and August 2016 and decided that it met the established criteria for elimination. The process included six years of work with countries to document evidence of the elimination [1].
